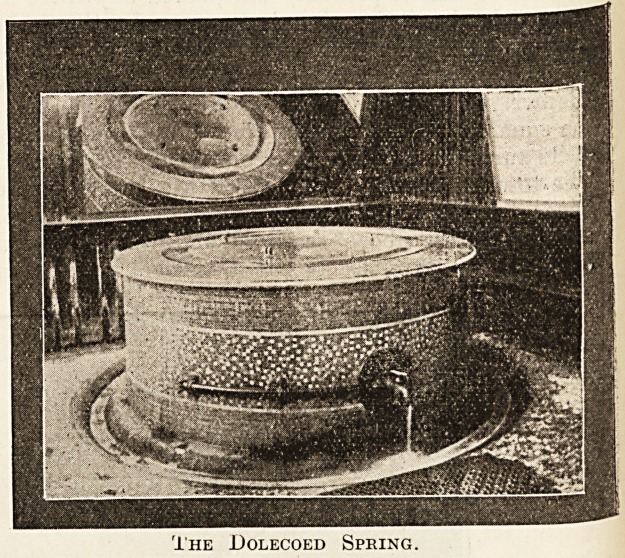# Home and Foreign Spas
*Previous articles of this series appeared in The Hospital of Jan. 28, Feb. 25, March 25, April 22, May 20, June 3, June 17, July 8, and July 29.


**Published:** 1911-08-19

**Authors:** 


### HOME AND FOREIGN SPAS.*

### X.—LLANWRTYD WELLS.

Although the natural advantages of Llanwrtyd Wells and the fame of its mineral waters in the treatment of various diseases are well known and appreciated throughout Wales, the Spa is practically unknown in England. It is one of a group of three Spas located in South Wales, and, whilst possessing merits probably unequalled anywhere in these islands, it is, singularly enough, the least known of the three outside the Principality. There is, however, unquestionably a "future" before Llanwrtyd, as the efficacy of its mineral springs, the equability of its climate, and the natural beauty of its surroundings become more widely recognised. The town is prettily situated on the banks of the River Irfon, in the northern part of Breconshire, some 800 feet above sea-level and well sheltered on all sides by hills. It has a resident population of about a thousand, is attractive, clean, and well lighted; the domestic water-supply is abundant and of excellent quality, and as there are no large factories in the neighbourhood the atmosphere is free from contamination, and, being tempered with invigorating breezes from the mountains, its purity and bracing effects greatly enhance the value of Llanwrtyd Wells as a health-resort.

### How Reached.

As the Spa is on the London and North Western main line to Swansea, there is a good train service to and from London (Euston), especially during the summer months. The distance from the Metropolis is two hundred and seventeen miles, and the journey is accomplished in six hours; through carriages are attached to most trains, so that the inconvenience of changing may be avoided. Llanwrtyd is also easily accessible from Cardiff, Swansea, and other chief centres, from which week-end and fortnightly tickets are issued.

### The Story of the Spa.

The curative properties of the springs of Llanwrtyd first became known in 1732. In that year the vicar of a neighbouring parish, being much troubled with an obstinate and ineradicable scurvy, was casually informed of the reputed poisonous spring known to the peasantry of the district as the Stinking Well. Curiosity led him to the spot, and whilst seated on the brink of the well he observed a frog evidently greatly enjoying the so-called venomous water. From this the reverend gentleman concluded that the water could not be poisonous, or the frog could not live there. Consequently, he ventured to drink of the spring, and, suffering no ill-effects, continued the treatment for a period of two months, drinking and bathing daily in the water of the well. In due time the scurvy disappeared, leaving the skin perfectly clear and free of disease, and it was from this experience that the fame of the sulphur water of Llanwrtyd Wells began, and spread over the Principality, gaining yet a greater reputation as the years passed and the record of relief obtained and cures effected became a gradually increasing one.

The River Irfon, Llanwrytd Wells.

### The Springs, Pump-Room, and Baths.

The Dolecoed Spring, situated in the centre of the picturesque Dolecoed Park, is the one to which Llanwrtyd Wells owes its origin. It is almost a pure sulphur water, the principal remedial agent being sulphuretted hydrogen, which is present to at least the amount of 10 cubic inches to the gallon. The source of the supply is practically inexhaustible; the water is neither pumped nor stored, but flows

* Previous articles of this series appeared in The Hospital of Jan. 28, Feb. 25, March 25, April 22, May 20, June 3, June 17, July 8, and July 29.

freely to the surface to the extent of some 4,500 gallons a day. To prevent the loss of gas by evaporation, the spring is hermetically sealed in a massive marble and mosaic circular pedestal covered with a disc of plate glass, through which the water can be seen in a state of effervescence as the gas rises to the surface. It is claimed that since the sealing of the well the sulphuretted hydrogen in the water has increased to 15 cubic inches per gallon.

An analysis of the water made by Professor Attfield gave the following result:—

| | |
|---|---|
| Chloride of potassium ... ... ... | 1.692 |
| Chloride of sodium ... ... ... | 60.782 |
| Chloride of magnesium ... ... ... | 0.871 |
| Chloride of calcium ... ... ... | 13.486 |
| Sulphate of calcium ... ... ... | 0.827 |
| Carbonate of calcium ... ... ... | 2.005 |
| Oxide of iron ... ... ... ... | 0.043 |
| Silica ... ... ... ... ... | 1.323 |
| Lithium, barium, bromine, iodine, and nitrates ... ... ... ... | *traces* |
| Total grains per gallon ... ... | 81.029 |
| Sulphuretted hydrogen, cubic inches ... | 10.0 |

(Specific gravity, 1.0011.)

The comfortable pump-room which has been built directly over the sulphur well also embraces within its wall a chalybeate spring, in which iron is present as ferrous carbonate to the extent of 1.522 grain to the gallon. The water is of a greenish-brown tint, quite odourless, and not unpleasant to drink.

The sulphur water is conveyed by gravitation from the sealed spring through three-inch ebonite pipes to the exceptionally well-fitted and comfortable baths in the Dolecoed Hotel, whilst every precaution is taken to ensure that it loses none of

THE DOLECOED PUMP-ROOM.

its gaseous and other constituents before use. The thermal apparatus employed in the bathing establishment is so excellent that it is possible to obtain baths at any temperature that may be prescribed without lessening in the smallest degree the therapeutic powers of the mineral water. The baths are not reserved to residents at the hotel, but are equally available to all who need them.

In addition to the Dolecoed Springs there are the Victoria Wells, discovered in 1897, on the opposite side of the River Irfon. They are four in number, and include a sulphur spring similar in composition to the old well, but not quite so strong; a sulphur magnesium spring; a mild lithia saline water containing, in addition to the usual saline constituents, 2.4 grains of lithium chloride and 1.16 grain of thallium chloride per gallon; and also a chalybeate spring, in which iron is present in the form of its carbonate. Attached to these wells

THE DOLECOED SPRING.

there is a bath-house, where sulphur and other baths may be had at very moderate charges.

### DISEASES TREATED.

The medicinal waters of Llanwrtyd, taken both internally and in the form of baths, have proved highly efficacious in the treatment of functional disorders of the liver, kidneys and stomach, gout, rheumatism, lumbago, sciatica and gall stones, all forms of skin disease, scrofula and glandular affections, chronic bronchitis, asthma, anæmia, nervous exhaustion, general debility, influenza, malaria, and other complaints induced by tropical climates.

### ACCOMMODATION AND AMUSEMENTS.

As Llanwrtyd Wells is essentially a health-resort, it is possible to secure excellent accommodation to suit all classes and means. There are two first-class hotels, the Dolecoed and the Abernant, fitted upon the most modern principles and replete with every comfort. In addition, in all parts of the town there are boarding and apartment houses, where board-residence may be obtained at very reasonable rates. The district provides ample opportunities for outdoor recreation, and sport of almost every description may be enjoyed. Tennis, croquet, and bowls have their grounds, whilst golfers are exceptionally well favoured, since there are two excellent courses, each of eighteen holes. Fishing is obtained in the River Irfon and its tributaries, rough shooting over the mountains and hills, and boating on the picturesque Abernant Lake. The scenery of the countryside is very beautiful, and there are many enjoyable walks in the neighbourhood, besides places of interest within driving distance of the Spa.

## Figures and Tables

**Figure f1:**
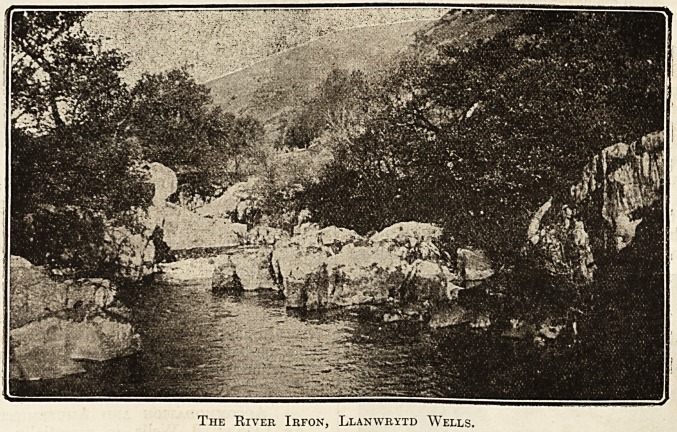


**Figure f2:**
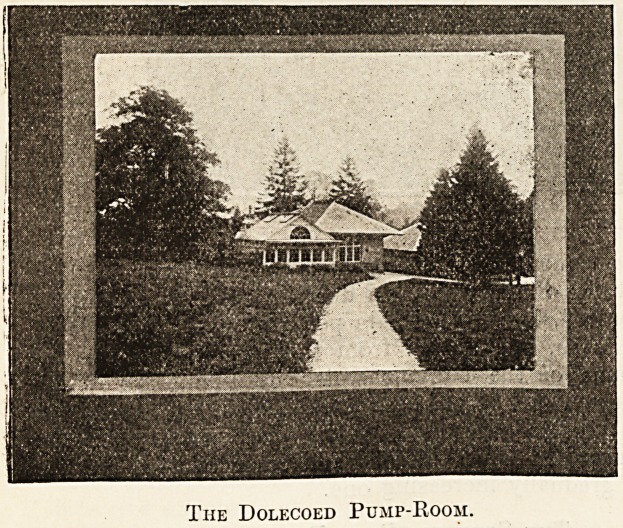


**Figure f3:**